# The relationship between maxillary and mandibular third molar germ stages and skeletal orthodontic malocclusions

**DOI:** 10.1093/ejo/cjag066

**Published:** 2026-09-08

**Authors:** Gönül Dinç, Hüseyin Melik Böyük, Saadet Çınarsoy Ciğerim

**Affiliations:** Department of Orthodontics, Faculty of Dentistry, Van Yuzuncu Yil University, Van 65080, Turkey; Department of Orthodontics, Faculty of Dentistry, Van Yuzuncu Yil University, Van 65080, Turkey; Department of Orthodontics, Faculty of Dentistry, Van Yuzuncu Yil University, Van 65080, Turkey

**Keywords:** third molar tooth germ, skeletal orthodontic malocclusion, Demirjian method

## Abstract

**Aim:**

To evaluate the relationship between third molar germ formation and three-dimensional (sagittal, vertical, and transverse) skeletal malocclusions to guide early orthodontic diagnosis and proactive treatment planning.

**Materials and Methods:**

A retrospective study was conducted using the initial orthodontic records of 483 patients (58.6% female, 41.4% male; mean age 12.42 ± 2.26 years) treated between 2014 and 2025. Initial panoramic, posteroanterior (PA), and lateral cephalometric radiographs were evaluated to determine Sella-Nasion-Point A Angle (SNA), Sella-Nasion-Point B Angle (SNB), Point A-Nasion-Point B Angle (ANB), and Sella-Nasion to Gonion-Gnathion Plane Angle (SN-GoGN) angles, along with maxillary-mandibular skeletal widths. Sagittal classification used ANB angle thresholds (Class III: ANB <0°; Class I: 0°–4°; Class II: ANB >4°); vertical classification used SN-GoGn (<27° = hypodivergent; 27°–38° = normodivergent; >38° = hyperdivergent); transverse classification used PA cephalometric skeletal widths relative to age- and sex-adjusted reference norms. Third molar germs were scored utilizing the Demirjian method. Inclusion criteria strictly required the absence of systemic diseases and prior third molar region surgeries.

**Results:**

In the early mixed dentition group, the germ formation stage of the mandibular right third molar (tooth 48) showed a nominally significant difference across sagittal skeletal malocclusion classes (*P* = .018; uncorrected). Skeletal Class III cases exhibited more advanced germ formation for tooth 48 compared to Skeletal Class II cases. However, following Bonferroni correction for multiple comparisons (corrected threshold *P* < .0028), this finding did not reach statistical significance and should be considered an exploratory observation. No significant differences were found regarding transverse or vertical skeletal malocclusions across any dentition groups (*P* > .05).

**Conclusions:**

(i) No significant correlation exists between maxillary/mandibular transverse skeletal widths and third molar germ formation stages. (ii) Skeletal Class III patients in the early mixed dentition period demonstrate accelerated development of tooth 48, likely reflecting advanced sagittal mandibular growth.

## Introduction

Tooth eruption is a physiological and developmental process where the developing tooth moves in three directions and expands within the dentoalveolar structure before active eruption [1]. To become functional, tooth germs must move toward the occlusal plane through complex stages. This process is divided into three periods: the pre-eruptive period (germ development within the bone), the eruptive period (rapid movement through the gingiva toward the occlusal plane), and the posteruptive period (adaptation to the occlusal plane alongside jaw development) [2]. Eruption timing varies based on dietary habits, tooth function, and racial or genetic characteristics; among these, third molars are the last to erupt [1].

Third molars frequently complicate comprehensive orthodontic treatment planning due to their highly variable formation, calcification, morphology, and eruption patterns. Their development and interaction with other teeth have long been a major concern in dentistry and orthodontics [2, 3]. These teeth typically appear on radiographs between ages 5 and 16 and usually erupt between ages 18 and 24 [4]. Massler *et al*. [5] reported that third molar germ formation begins at 3–4 years, calcification at 7–10 years, crown calcification at 12–16 years, and eruption at 17–21 years. While often not directly involved in orthodontic mechanics, they frequently require inclusion in treatment planning.

While the etiology of third molar impaction is multifactorial, it is fundamentally linked to craniofacial growth patterns, genetic predispositions, and available retromolar space. During growth, skeletal changes and the direction of bone growth significantly affect dental positions [1]. Skeletal orthodontic anomalies arise from the disruption of the relationship between the jawbones and the skull [6]. The likelihood of third molar eruption is a critical consideration for treatment planning and long-term stability, making it vital for dentists and orthodontists [7]. Broadbent [8] suggested that failure of the mandible to reach full growth potential causes impaction, while Begg [9] attributed impaction to insufficient forward movement of the teeth. Furthermore, third molars can hinder orthodontic treatment or cause distal molar movement [10–12].

In modern societies, third molar impaction is significantly more common than in other teeth [4], often due to inadequate retromolar space in small jaws [13–15]. Regardless of etiology, impaction may relate to facial growth patterns [16, 17]. Despite their profound clinical impact, there is a notable scarcity of literature examining the direct relationship between third molar germ developmental stages and three-dimensional (sagittal, vertical, and transverse) skeletal malocclusions. Therefore, this study aims to evaluate these relationships to serve as a robust diagnostic guide for early orthodontic intervention, addressing a significant gap in the literature.

## Materials and methods

**Figure 1:**
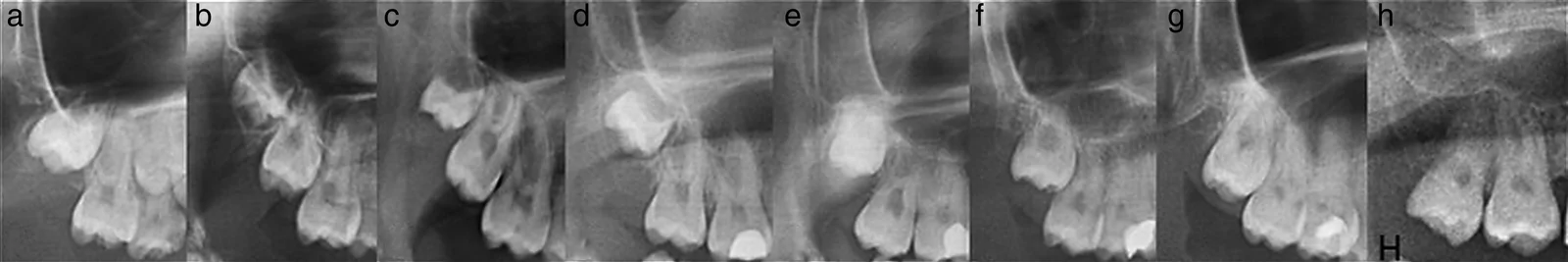
Classification of the upper right third molar teeth of the patients in the study according to the Demirjian method.

This retrospective study analyzed the initial orthodontic records of 483 patients (283 females, 200 males; mean age 12.42 ± 2.26 years, range 5.9–15.9) referred to the Orthodontics Department between 2014 and 2025. Initial panoramic images, and posteroanterior and lateral cephalometric radiographs of the patients were evaluated. Cephalometric analyses determined Sella-Nasion-Point A Angle (SNA), Sella-Nasion-Point B Angle (SNB), Point A-Nasion-Point B Angle (ANB), Sella-Nasion to Gonion-Gnathion Plane Angle (SN-GoGN) angles and maxillary-mandibular skeletal widths. In panoramic images, third molar germs were scored according to the Demirjian method (Fig. 1).

Patients without chronic, systemic, or genetic diseases, growth and development disorders, and who had not undergone any surgical operation involving the third molar region were included in the study. Patients with pathological conditions affecting the third molar teeth and unacceptable radiographic image quality were excluded from the study. The study protocol was approved by the Non-Interventional Clinical Studies Ethics Committee (Decision no: 2025/10-38) and conducted in strict adherence to the Declaration of Helsinki.

The calcification development of the third molar teeth was classified into eight stages (A–H) according to the Demirjian method; for clarity, these Demirjian calcification stages are referred to as germ developmental stages throughout the manuscript. This method includes the first four levels (A–D) covering the period from the beginning of calcification in the tubercles to the completion of crown formation and the last four levels (E–H) covering the period from the formation of bifurcation to root formation and apical occlusion. Massler *et al*. [5] reported that crypt formation for third molar teeth begins at 3 to 4 years of age. It is known that calcification begins at 7–10 years, crown calcification is completed at 12–16 years, and eruption begins between 17 and 21 years of age. Because third molar extractions due to localized pathologies (e.g. pericoronitis) are highly improbable before the age of 15, the upper age limit for inclusion was set at 15 years and 11 months. For dentition groups, early mixed dentition (6–8.99 years), late mixed dentition (9–11.99 years), and permanent dentition (12–15.99 years) groups were created.

Skeletal sagittal classification was based on the ANB angle: ANB <0° was classified as Skeletal Class III, 0° ≤ANB ≤4° as Skeletal Class I, and ANB >4° as Skeletal Class II. Vertical skeletal classification was determined by the SN-GoGn angle: SN-GoGn <27° was categorized as hypodivergent (low angle), 27°–38° as normodivergent (norm angle), and SN-GoGn >38° as hyperdivergent (high angle). Transverse skeletal dimensions were assessed from posteroanterior (PA) cephalometric radiographs using the maxillary and mandibular skeletal width measurements described by Ricketts *et al*. Widths more than one standard deviation below the age- and sex-adjusted reference mean were classified as narrow, within ±1 SD as normal, and more than one standard deviation above the reference mean as wide. The elevated proportion of subjects classified as having a narrow maxilla (69.6%) reflects the nature of the orthodontic referral population: patients presenting for orthodontic consultation disproportionately exhibit transverse skeletal deficiencies, and this prevalence is consistent with figures reported in comparable orthodontic cohort studies.

An *a priori* power analysis using G*Power (v3.1.7), based on the developmental measurements reported by Tozar *et al*. [18] determined that a minimum of 150 participants was required to achieve 80% power at an *α* = 0.05 level (effect size *d* = 0.461).

### Statistical analysis

Statistical analyses were performed utilizing the SPSS 27 program. Descriptive statistics such as mean, standard deviation, median, minimum and maximum values, frequency, and percentage were calculated. The Shapiro-Wilks test, skewness and kurtosis values, and Box Plot graphs were used to assess the suitability of the data to a normal distribution.

Because the variables did not exhibit a normal distribution, the Kruskal–Wallis test was employed for multi-group comparisons, followed by the Dunn test to isolate group differences. Spearman’s correlation analysis was performed to evaluate the relationships between variables. Statistical significance was defined as *P* < .05 within a 95% confidence interval. Given that multiple statistical comparisons were performed across four third molar positions, three dentition groups, and three skeletal classification dimensions (sagittal, vertical, and transverse), the potential for type I error inflation was considered. To address this, the Bonferroni correction was applied, yielding a corrected significance threshold of *P* < .0028 (i.e. 0.05/18 independent comparisons). The sole finding of *P* = .018 (tooth 48, early mixed dentition, sagittal classification) did not reach the Bonferroni-corrected threshold; accordingly, this result should be interpreted as an exploratory, hypothesis-generating finding. Future confirmatory studies with larger, adequately powered samples are warranted to validate this observation.

Because the dentition subgroups were unequal in size, a *post hoc* (observed) power analysis was additionally performed for the early mixed dentition subgroup (*n* = 42; Class I = 14, Class II = 16, Class III = 12), which was the smallest subgroup and the only one yielding a nominal association (Supplementary Table S1). For each Kruskal–Wallis comparison, the effect size was expressed as epsilon-squared (*ε*^2^), and both the 95% confidence interval for *ε*^2^ and the achieved power at *α* = 0.05 were estimated by Monte Carlo resampling (20 000 iterations) from the observed group distributions. For tooth 48, the observed effect was large (*ε*^2^ = 0.20; 95% CI, 0.04–0.50) and the achieved power was 75%, indicating that the nominal association (*P* = .018) was unlikely to represent a spurious result. For the remaining third molars, the observed effects were small (*ε*^2^ = 0.01–0.06) and the corresponding achieved power was low (9%–27%). A sensitivity analysis confirmed that, at this subgroup size, the power to detect a conventionally small effect (*ε*^2^≈0.04) was ∼4%, and ∼27% for a medium effect (*ε*^2^≈0.10). Accordingly, the nonsignificant findings within the smaller subgroups are interpreted as reflecting limited statistical power rather than definitive evidence of no association.

## Results

The ages of the participants in the study ranged from 5.9 to 15.9 years, with an average of 12.42 ± 2.26. The total number of evaluated maxillary third molars was 746 and mandibular third molars was 808. The number of patients lacking all teeth (18, 28, 38, and 48) was 43. The number of individuals with teeth (38 and 48) but lacking teeth (18 and 28) was 34. The number of patients with teeth (18 and 28) but lacking teeth (38 and 48) was 20. The number of patients lacking only tooth (18) was 30, only tooth (28) was 19, only tooth (38) was 6, and only tooth (48) was 7. The demographic distribution comprised 8.7% (*n* = 42) in the early mixed dentition stage, 33.7% (*n* = 163) in the late mixed dentition stage, and 57.6% (*n* = 278) in the permanent dentition stage. Descriptive cephalometric values for SNA, SNB, ANB, and SN-GoGn are presented in Table 1.

**Table 1:** Distribution of cephalometric characteristics.

		*n* (%)
SNA	*Mean* *±* *SD*	79.48 ± 3.82
	*Median (Min–Max)*	79.8 (67.8–93)
SNB	*Mean* *±* *SD*	76.6 ± 4.05
	*Median (Min–Max)*	76.4 (64–91.7)
ANB	*Mean* *±* *SD*	2.92 ± 3.18
	*Median (Min–Max)*	3 (−7–14.5)
SN-GoGn	*Mean* *±* *SD*	34.27 ± 5.49
	*Median (Min–Max)*	34.1 (16.2–60)
According to point A	**Retrognathic maxilla**	251 (52)
	**Ortognathic maxilla**	184 (38.1)
	**Prognathic maxilla**	48 (9.9)
According to point B	**Retrognathic mandible**	305 (63.1)
	**Ortognathic mandible**	132 (27.3)
	**Prognathic mandible**	46 (9.5)

When the distribution of cases according to skeletal malocclusions was examined, it was seen that 48% (*n* = 232) of the cases were class 1, 35.2% (*n* = 170) were class 2, and 16.8% (*n* = 81) were class 3 in the sagittal direction. When vertical skeletal evaluation was performed, 8.1% (*n* = 39) of the cases were low angle, 67.5% (*n* = 326) were norm angle, and 24.4% (*n* = 118) were high angle. When transverse skeletal evaluation was performed, it was observed that 69.6% (*n* = 336) of the cases had a narrow maxilla, 26.3% (*n* = 127) had a normal maxilla, and 4.1% (*n* = 20) had a wide maxilla. In the mandible evaluation, 56.9% (*n* = 275) had a narrow mandible, 27.1% (*n* = 131) had a normal mandible, and 15.9% (*n* = 77) had a wide mandible. The distribution of skeletal malocclusion classifications is summarized in Table 2.

**Table 2:** Distribution of skeletal classifications.

		*n* (%)
Skeletal class	**1**	232 (48.0)
	**2**	170 (35.2)
	**3**	81 (16.8)
Vertical class	**Low angle**	39 (8.1)
	**Normal angle**	326 (67.5)
	**High angle**	118 (24.4)
Maxillary class	**Narrow maxilla**	336 (69.6)
	**Normal maxilla**	127 (26.3)
	**Wide maxilla**	20 (4.1)
Width (PA)	*Mean* *±* *SD*	59.68 ± 6.35
	*Median (Min–Max)*	60.3 (33.9–83.4)
Mandibular class	**Narrow mandible**	275 (56.9)
	**Normal mandible**	131 (27.1)
	**Wide mandible**	77 (15.9)
Width (PA)	*Mean* *±* *SD*	79.89 ± 9.09
	*Median (Min–Max)*	79.9 (41.1–118.3)

**Figure 2:**
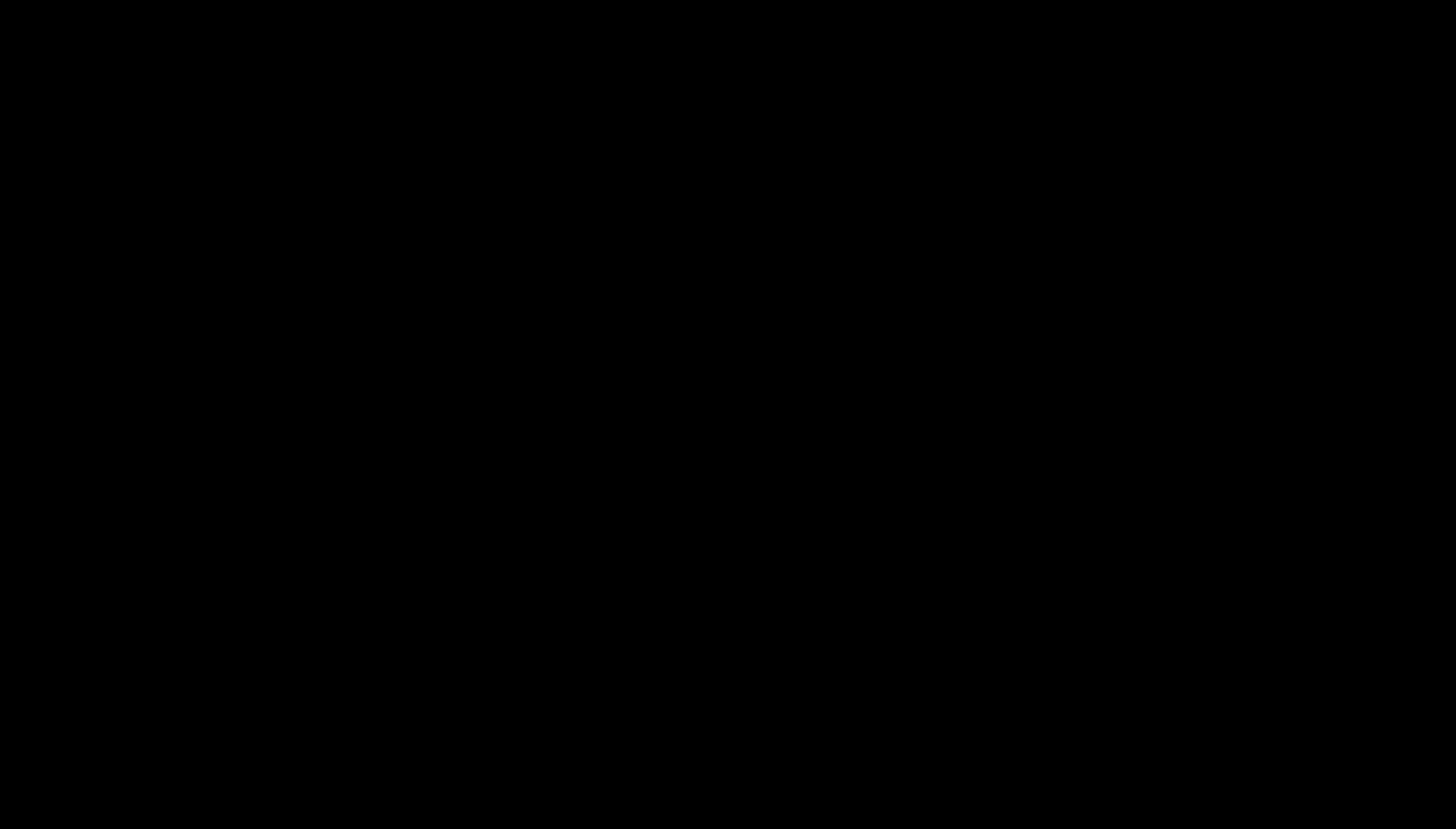
Distribution of 48 germ stages according to skeletal classes.

The distribution of the germ development stages of teeth numbered 18, 28, 38, and 48 is shown in Table 3. In the early mixed dentition group, germ formation stages 18, 28, and 38 did not show statistically significant differences according to sagittal skeletal malocclusion classes (*P* > .05). However, the developmental stage of tooth 48 displayed a nominally significant difference (*P* = .018; uncorrected), with Skeletal Class III patients exhibiting more advanced germ formation compared to Class II patients (Fig. 2). This finding did not reach the Bonferroni-corrected significance threshold (*P* < .0028) and is therefore interpreted as an exploratory observation.

**Table 3:** Distribution of germ developmental stages.

	18 germ stage	28 germ stage	38 germ stage	48 germ stage
0	114 (23.6)	106 (21.9)	78 (16.1)	80 (16.6)
1	54 (11.2)	54 (11.2)	67 (13.9)	52 (10.8)
2	36 (7.5)	33 (6.8)	34 (7)	37 (7.7)
3	44 (9.1)	53 (11)	83 (17.2)	92 (19)
4	129 (26.7)	132 (27.3)	129 (26.7)	130 (26.9)
5	63 (13)	65 (13.5)	50 (10.4)	48 (9.9)
6	29 (6)	28 (5.8)	30 (6.2)	31 (6.4)
7	13 (2.7)	9 (1.9)	12 (2.5)	11 (2.3)
8	1 (0.2)	3 (0.6)	0 (0.0)	2 (0.4)
*Mean* *±* *SD*	2.82 ± 2.11	2.87 ± 2.07	2.93 ± 1.93	2.98 ± 1.93
*Median (Min–Max)*	3 (0–8)	3 (0–8)	3 (0–7)	3 (0–8)

In both the late mixed and permanent dentition cohorts, no statistically significant differences in germ formation stages were detected for any third molar across sagittal skeletal classes (*P* > .05) (Table 4). Furthermore, germ development stages for all evaluated third molars (18, 28, 38, 48) showed no statistically significant differences across vertical or transverse skeletal malocclusion classes in any dentition group (*P* > .05) (Tables 5 and 6).

**Table 4:** Comparison of germ stages according to skeletal classes.

		Skeletal class			*P*
		Class 1	Class 2	Class 3	
**Early mixed dentition period**		***n* = 14**	***n* = 16**	***n* = 12**	
18 germ stage	*Mean* *±* *SD*	0.43 ± 0.76	0.31 ± 0.6	0.83 ± 1.47	***0.583* ^a^**
	*Median (Min–Max)*	0 (0–2)	0 (0–2)	0 (0–5)	
28 germ stage	*Mean* *±* *SD*	0.43 ± 0.65	0.31 ± 0.6	0.75 ± 1.48	***0.780* ^a^**
	*Median (Min–Max)*	0 (0–2)	0 (0–2)	0 (0–5)	
38 germ stage	*Mean* *±* *SD*	0.50 ± 0.65	0.38 ± 0.5	1.17 ± 1.53	***0.290* ^a^**
	*Median (Min–Max)*	0 (0–2)	0 (0–1)	1 (0–5)	
48 germ stage	*Mean* *±* *SD*	0.43 ± 0.65	0.25 ± 0.45	1.25 ± 1.42	*0.018* ^a,^ *
	*Median (Min–Max)*	0 (0–2)	0 (0–1)	1 (0–5)	
**Late mixed dentition period**		***n* = 78**	***n* = 58**	***n* = 27**	
18 germ stage	*Mean* *±* *SD*	2.22 ± 1.66	2.05 ± 1.63	1.48 ± 1.67	***0.117* ^a^**
	*Median (Min–Max)*	2 (0–6)	2 (0–5)	1 (0–5)	
28 germ stage	*Mean* *±* *SD*	2.19 ± 1.74	2.1 ± 1.54	1.59 ± 1.62	***0.338* ^a^**
	*Median (Min–Max)*	2 (0–7)	2 (0–5)	1 (0–4)	
38 germ stage	*Mean* *±* *SD*	2.23 ± 1.37	1.98 ± 1.52	2 ± 1.44	***0.570* ^a^**
	*Median (Min–Max)*	2.5 (0–6)	2 (0–5)	2 (0–4)	
48 germ stage	*Mean* *±* *SD*	2.29 ± 1.42	2.16 ± 1.45	2.15 ± 1.46	***0.828* ^a^**
	*Median (Min–Max)*	3 (0–6)	3 (0–5)	3 (0–4)	
**Permanent dentition period**		***n* = 140**	***n* = 96**	***n* = 42**	
18 germ stage	*Mean* *±* *SD*	3.64 ± 1.95	3.51 ± 2.1	3.88 ± 2.15	***0.479* ^a^**
	*Median (Min–Max)*	4 (0–8)	4 (0–7)	4 (0–7)	
28 germ stage	*Mean* *±* *SD*	3.67 ± 1.85	3.55 ± 2.04	4.19 ± 1.92	***0.174***
	*Median (Min–Max)*	4 (0–8)	4 (0–7)	4 (0–8)	
38 germ stage	*Mean* *±* *SD*	3.91 ± 1.63	3.38 ± 1.92	4.1 ± 1.96	***0.090* ^a^**
	*Median (Min–Max)*	4 (0–7)	4 (0–6)	4 (0–7)	
48 germ stage	*Mean* *±* *SD*	3.86 ± 1.71	3.50 ± 1.89	4.21 ± 1.91	***0.072* ^a^**
	*Median (Min–Max)*	4 (0–8)	4 (0–7)	4 (0–8)	

Bold values indicate statistically significant differences. *indicates statistically significant.

^a^Kruskal–Wallis test and Dunn–Bonferroni test.

**Table 5:** Comparison of germ stages according to vertical classes.

		Vertical classes			*P*
		Low angle	Normal angle	High angle	
**Early mixed dentition period**		***n* = 2**	***n* = 29**	***n* = 11**	
18 germ stage	*Mean* *±* *SD*	0.00 ± 0.00	0.62 ± 1.08	0.27 ± 0.65	***.336* ^a^**
	*Median (Min–Max)*	0 (0–0)	0 (0–5)	0 (0–2)	
28 germ stage	*Mean* *±* *SD*	0.00 ± 0.00	0.55 ± 1.06	0.36 ± 0.67	***.584* ^a^**
	*Median (Min–Max)*	0 (0–0)	0 (0–5)	0 (0–2)	
38 germ stage	*Mean* *±* *SD*	0.50 ± 0.71	0.76 ± 1.12	0.36 ± 0.5	***.652* ^a^**
	*Median (Min–Max)*	0.5 (0–1)	0 (0–5)	0 (0–1)	
48 germ stage	*Mean* *±* *SD*	1.00 ± 0.00	0.69 ± 1.11	0.27 ± 0.47	***.191* ^a^**
	*Median (Min–Max)*	1 (1–1)	0 (0–5)	0 (0–1)	
**Late mixed dentition period**		***n* = 7**	***n* = 113**	***n* = 43**	
18 germ stage	*Mean* *±* *SD*	2.71 ± 2.43	2.01 ± 1.63	2.00 ± 1.63	***.730* ^a^**
	*Median (Min–Max)*	3 (0–6)	2 (0–6)	2 (0–5)	
28 germ stage	*Mean* *±* *SD*	2.14 ± 1.95	2.05 ± 1.69	2.07 ± 1.56	***.985* ^a^**
	*Median (Min–Max)*	2 (0–5)	2 (0–7)	2 (0–5)	
38 germ stage	*Mean* *±* *SD*	3.29 ± 1.38	2.04 ± 1.42	2.09 ± 1.41	***.171* ^a^**
	*Median (Min–Max)*	3 (2–6)	2 (0–5)	2 (0–4)	
48 germ stage	*Mean* *±* *SD*	3.00 ± 1.83	2.19 ± 1.41	2.19 ± 1.42	***.532* ^a^**
	*Median (Min–Max)*	3 (0–6)	3 (0–5)	3 (0–4)	
**Permanent dentition period**		***n* = 30**	***n* = 184**	***n* = 64**	
18 germ stage	*Mean* *±* *SD*	3.2 ± 2.19	3.77 ± 1.88	3.44 ± 2.34	***.529* ^a^**
	*Median (Min–Max)*	4 (0–7)	4 (0–8)	4 (0–7)	
28 germ stage	*Mean* *±* *SD*	3.47 ± 2.01	3.8 ± 1.82	3.56 ± 2.2	***.757* ^a^**
	*Median (Min–Max)*	4 (0–7)	4 (0–8)	4 (0–8)	
38 germ stage	*Mean* *±* *SD*	3.5 ± 2.13	3.9 ± 1.69	3.47 ± 1.94	***.333* ^a^**
	*Median (Min–Max)*	4 (0–7)	4 (0–7)	4 (0–7)	
48 germ stage	*Mean* *±* *SD*	3.53 ± 2.16	3.92 ± 1.68	3.55 ± 1.98	***.538* ^a^**
	*Median (Min–Max)*	4 (0–7)	4 (0–8)	4 (0–8)	

Bold values indicated statically significant differences.

^a^Kruskal–Wallis test.

**Table 6:** The relationship between maxillary width, mandibular width, and germ stages.

		Maxillary width	Mandibular width
**Early mixed dentition period**			
18 germ stage	***r***	0.105	—
	***P***	***.507***	—
28 germ stage	***r***	0.094	—
	***P***	***.555***	—
38 germ stage	***r***	—	0.184
	***P***	—	***.242***
48 germ stage	***r***	—	0.087
	***P***	—	***.586***
**Late mixed dentition period**			
18 germ stage	***r***	0.080	—
	***P***	***.309***	—
28 germ stage	***r***	0.078	—
	***P***	***.324***	—
38 germ stage	***r***	—	0.140
	***P***	—	***.075***
48 germ stage	***r***	—	0.130
	***P***	—	***.097***
**Permanent dentition period**			
18 germ stage	***r***	−0.009	—
	***P***	***.888***	—
28 germ stage	***r***	0.026	—
	***P***	***.661***	—
38 germ stage	***r***	—	0.041
	***P***	—	***.496***
48 germ stage	***r***	—	0.049
	***P***	—	***.411***

Bold values indicated statically significant differences.

*r*, Spearman’s correlation coefficient.

## Discussion

Impacted third molars represent a ubiquitous clinical challenge in orthodontic practice, driven by a multifactorial etiology. The mechanistic question of interest in the present study concerns the interaction between jaw growth and germ development: as the mandible grows sagittally and vertically, the retromolar region remodels, and the timing and direction of this remodeling are thought to interact with the developmental schedule of the third molar germ. Genetic factors that govern jaw dimension have been proposed as a shared upstream determinant of both processes [19, 20]. Building on this mechanistic framework, the studies discussed below are contrasted by the specific skeletal dimension they examined and by their methodology, rather than grouped together. Sagittal malocclusions heavily influence third molar positioning; Skeletal Class II cases often present with posterior mandibular spatial deficiencies, predisposing third molars to oblique or mesioangular impactions [21]. In Skeletal Class III individuals, mandibular width can somewhat improve the impacted molar position, which can increase the eruption potential [22]. Recent cone-beam computed tomography analyses have robustly corroborated these mechanisms, demonstrating significant correlations between mandibular arch length, intercanine width, and the depth of impacted molars.

The aim of this study is to evaluate the relationship between third molar tooth germ formation and different types of skeletal malocclusion to guide early diagnosis and ensure more effective treatment outcomes with appropriate timing. Tooth development is one of the most reliable methods for determining chronological age [23]. This study utilized the Demirjian method due to its proven reliability, simplicity, and low inter-observer variability. Hagg *et al*. [24] previously validated that this method yields superior accuracy in younger cohorts compared to adults. While panoramic radiographs carry inherent two-dimensional limitations, they remain an optimal and universally adopted diagnostic tool for simultaneously assessing the maxillomandibular complex and broad dental age [25].

Sato [26] posited that posterior crowding induced by third molars actively distorts dentofacial skeletal structures and the development of malocclusions. Massler *et al*. [5] reported that crypt formation begins at 3–4 years, calcification at 7–10 years, and eruption at 17–21 years. Therefore, in our study, patients under the age of 16 were selected, with an average age of 12.42 ± 2.26. Ricketts [27] emphasized that eruption direction plays a critical role in impaction, while Kaplan [21] associated impaction with larger mandibular growth angles. Richardson’s [19] longitudinal data further highlighted that Skeletal Class II mandibles with reduced dimensions are highly prone to third molar impaction. Because our study strictly evaluated individuals under 16 years of age, physical impaction was not the primary measured outcome; instead, our unique focus was entirely on the early developmental stages of the tooth germs.

Farzanegan and Goya [28] reported that retromolar spacing varies significantly among vertical growth patterns, observing maximum spacing in normal vertical growth patterns. This confirmed Kaplan’s findings regarding insufficient ramus resorption in skeletal deep bite. However, Mollaoglu *et al*. [29] reported the angle between the third molar and the mandibular base is larger when the retromolar space is smaller. Unlike previous studies focusing almost exclusively on retromolar space availability, our investigation found no significant relationship between vertical skeletal malocclusions and third molar germ developmental stages across any dentition group. This divergence in findings likely originates from our novel methodological approach, which categorized patients by active germ development rather than terminal impaction status.

To the best of our knowledge, this is the first study to comprehensively correlate third molar germ developmental stages directly with simultaneous sagittal, vertical, and transverse skeletal malocclusions. The literature explicitly linking early third molar bud formation to definitive facial structures remains sparse. One study reported that the reason Skeletal Class III patients have fewer third molar germs than Skeletal Class II patients is unclear, though polygenetic inheritance may be associated with genes controlling jaw dimensions.

Kajii *et al*. [20] previously investigated Japanese orthodontic patients, noting the percentage of Skeletal Class II patients with third molar teeth was higher than that of Skeletal Class III patients. They also reported the frequency of mandibular third molar germs to be higher than maxillary ones. Unlike Kajii *et al*. [20], who merely evaluated the presence or absence of these germs, the present study quantified precise developmental stages. Consistent with demographic trends, we found mandibular germs (808) to be more frequent than maxillary ones (746). In the present study, no relationship was found between the transverse skeletal dimension (maxillary or mandibular width) and third molar germ developmental stage in any dentition group. The only divergent observation concerned the sagittal dimension: in the early mixed dentition group, tooth 48 showed nominally more advanced development in Skeletal Class III than in Skeletal Class II patients (*P* = .018, uncorrected), a finding that did not survive Bonferroni correction and is therefore regarded as exploratory.

Vertical skeletal patterns undeniably influence eventual impaction; hyperdivergent alveolar development often leads to upright positioning and delayed eruption [30, 31]. In hypodivergent individuals, compact bone structure and limited posterior space can increase the likelihood of impaction [22]. Transversely, arch discrepancies severely constrict posterior capacity and increase impaction risk [22]. A 2024 retrospective study by Peña-Reyes *et al*. reported no significant difference in mandipular thir molar angulation between extraction and non-extraction orthodontic treatment groups, indicating that premolar extraction alone may not reliably predict improved third molar eruption [32]. Our findings supplement this by demonstrating that third molar development is not merely a function of retromolar space but is intricately tied to early underlying skeletal malocclusion types and developmental angulations.

This study presents several limitations. First, the cross-sectional design inherently precludes the assessment of time-dependent developmental changes and eruption trajectories, restricting conclusions to correlations rather than causations. A further limitation concerns the unequal distribution of subjects across dentition groups; the early mixed dentition subgroup (*n* = 42) was substantially smaller than the permanent dentition subgroup (*n* = 278). *Post hoc* analysis indicated that this subgroup was adequately powered to detect the large effect observed for tooth 48 (power = 75%) but was underpowered for small-to-moderate effects (power <30%). Consequently, the null findings within the smaller subgroups should be interpreted as an absence of a detectable association rather than as definitive evidence of no relationship, and require confirmation in larger, balanced samples. Second, while panoramic radiography provides a broad anatomical overview, its two-dimensional nature prevents the precise three-dimensional spatial and buccolingual assessment of the developing germs. Finally, critical variables such as genetic inheritance, environmental influences, and functional soft-tissue habits were not evaluated.

## Conclusion

1. No statistically significant correlation exists between maxillary or mandibular transverse skeletal widths and third molar germ formation stages.
2. Skeletal Class III patients in the early mixed dentition period demonstrate accelerated developmental stages of the mandibular right third molar (tooth 48) compared to Skeletal Class II patients.
3. It is hypothesized that the exaggerated sagittal growth of the mandible inherent to Class III malocclusions actively accelerates the early development of the lower third molars. Further studies should be conducted to investigate maxillary and mandibular masses associated with third molar germs, alongside the molecular genetics of craniofacial development.

## Supplementary Material

cjag066_Supplementary_Data

## Data Availability

The datasets used and/or analyzed during the current study are available from the corresponding author on reasonable request.
