# Supplementary material for: The relationship between maxillary and mandibular third molar germ stages and skeletal orthodontic malocclusions
Source: Eur J Orthod. 2026 Sep 8;48(5):cjag066. doi: 10.1093/ejo/cjag066 (PMC13552500; doi:10.1093/ejo/cjag066)
Supplement: Supplementary file 1 [file cjag066_supplementary_data.docx]

**Supplementary Table S1: Post-hoc power analysis of the early mixed dentition subgroup**

Kruskal–Wallis comparisons of third molar germ developmental stage across sagittal skeletal classes (Class I/II/III). Early mixed dentition subgroup; n=42 (Class I=14, Class II=16, Class III=12).

| **Tooth (FDI)** | **H statistic** | **p value** | **Effect size (ε²)** | **95% CI (ε²)** | **Observed power** | **Interpretation** |
| --- | --- | --- | --- | --- | --- | --- |
| 18 | 1.080 | 0.583 | 0.026 | 0.002–0.254 | 13.7% | No association; underpowered |
| 28 | 0.497 | 0.780 | 0.012 | 0.002–0.215 | 9.0% | No association; underpowered |
| 38 | 2.472 | 0.291 | 0.060 | 0.004–0.321 | 26.9% | No association; underpowered |
| **48** | 8.007 | **0.018** | **0.195** | 0.043–0.499 | **75.3%** | Nominal association; adequately powered |

*FDI, Fédération Dentaire Internationale tooth notation; CI, confidence interval; ε², epsilon-squared.*

*Effect size (ε²) was derived from the Kruskal–Wallis H statistic as ε² = H(n+1)/(n²−1); values of approximately 0.01, 0.06 and 0.14 denote small, medium and large effects, respectively.*

*The 95% confidence intervals for ε² and the observed power were estimated by Monte Carlo resampling (20,000 iterations) from the observed group distributions, at α=0.05.*

*Sensitivity analysis (n=42): the power to detect a conventionally small effect (ε²≈0.04) was approximately 4%, and approximately 27% for a medium effect (ε²≈0.10).*

*For tooth 48, Dunn's post-hoc test (Bonferroni-adjusted) confirmed a significant pairwise difference between Class III and Class II (p=0.017); the mean germ developmental stage was 1.25 (Class III) versus 0.25 (Class II).*
